# Further Evidence that Onabotulinum Toxin is a Viable Treatment Option for Pediatric Chronic Migraine Patients

**DOI:** 10.7759/cureus.4343

**Published:** 2019-03-29

**Authors:** Sameer S Ali, Ilya Bragin, Elizabeth Rende, Luis Mejico, Klaus E Werner

**Affiliations:** 1 Neurology, Veterans Affairs Connecticut Healthcare System, West Haven, USA; 2 Neurology, St. Lukes University Health Network, Bethlehem, USA; 3 Neurology, Duke University, Durham, USA; 4 Neurology, State University of New York, Syracuse, USA

**Keywords:** chronic migraine, pain management, pediatrics, children, headaches, stress, social health, academic performance, family relations, pediatric migraine

## Abstract

Introduction

Chronic migraine is particularly devastating. It affects school work, extracurricular activities, and quality of life, including relationships with other family members, and can also influence the mental health of both the migraineurs and family members. According to the International Classification of Headache Disorders, 3rd edition (ICHD-3), chronic migraine is defined as 15 or more headache days per month for greater than three months, where at least on eight days per month, there are features of migraine headache. Although botulinum toxin type A (BoNTA) has been proven effective for treating chronic migraine in adults, little literature exists about its use in children. Here, we present the treatment response in children with chronic migraines treated with BoNTA at our institutions Duke and State University of New York (SUNY) Upstate.

Method

A retrospective analysis of 30 adolescent migraineurs who met ICHD-3 criteria for chronic migraine were treated with BoNTA injection according to the standardized adult protocol. Descriptive statistics and paired t-tests were performed. A total of 185 units of botulinum toxin were injected intramuscularly per patient, as in addition to the standard 31 sites for a total of 155 units, an additional 30 units were given in areas that were felt to provide further benefit.

Results

Participants (n=30) were 16.5 ± 1.83 years old. The headaches were precipitated by trauma in seven cases. All had failed standard pharmacotherapy, including amitriptyline and topiramate. An average of 2.47 ± 1.6 BoNTA injection cycles was performed. Migraine severity decreased significantly from 7.47 ± 1.89 on a 10-point scale to 4.34 ± 3.02 (p<.001). Additionally, headache frequency improved from 24.4 ± 7.49 painful days per month to 14.8 ± 12.52 painful days per month (p<.001). One patient developed nausea related to injections; all others tolerated it well, with no side effects.

Discussion

BoNTA injection was a safe and effective therapy for chronic migraine in our cohort of children recalcitrant to medical therapy. Further research with multi-centered, double-blinded, randomized, placebo-controlled trials is warranted to evaluate the long-term safety and efficacy in this population.

## Introduction

Migraine in children is one of the most common neurologic conditions in pediatrics. It is a leading cause of disability across all age groups. It appears that the typical onset of migraine is during early to mid-adolescence. It can begin at any age and, unfortunately for many, this early onset of migraine often continues into adulthood [1-2].

Migraine headache in children and adolescents aged under 18 years is more often bilateral rather than the more typical unilateral pain seen in adults, which emerges in late adolescence or early adult life [3]. Prevalence varies amongst the current literature, however, it appears to occur in approximately 2%-6% of children [4-8]. According to one paper, there was a 3% prevalence in younger, school-age children, in line within the prevalence rate range described above; however, this shot up to 20% in older adolescents [9]. A migraine headache in children is usually frontotemporal and an occipital headache is rare and should be further evaluated. Clinical observation is critical in younger children, and photophobia and photophobia may be inferred from their behavior [3]. Despite these few differences in presentation between the adult and pediatric populations, overall, the clinical presentation of migraine is similar, regardless of age, and the pathophysiology is thought to be similar [10].

The International Classification of Headache Disorders, 3rd edition (ICHD-3) defines chronic migraine as 15 or more headache days per month for greater than three months and on at least eight days per month, there are features of migraine headache. The other days can also be migraine or tension-type; however, at least eight days per month have to be migrainous. Current literature regarding pediatric headaches uses terminology such as chronic migraine and chronic daily headache, including both terms in the few retrospective studies analyzing onabotulinum toxin use in pediatric patients [5-6,11-13]. For the purpose of clarity, chronic migraine and chronic tension type headaches are the most frequent subtypes of chronic daily headache, which itself may be primary or secondary, and, overall, headache disorders are very difficult to treat [4,14].

Although onabotulinum toxin Type A (BoNTA) has been proven effective for treating chronic migraine in adults, scant literature exists about its use in children, as evidenced by the only handful of retrospective studies published and cited above. Botulinum toxins are compounds derived from the bacterial species Clostridium botulinum. They are known to relax involuntarily spasming muscles by blocking the release of acetylcholine, the neurotransmitter that initiates muscle contraction at the neuromuscular junction [15]. They are a well-established class of neurotoxins with a wide variety of Food and Drug Administration (FDA)-approved medical applications, including muscle relaxation in cervical dystonia, headache prevention, and spastic bladder control [16]. In addition to the muscle relaxation effects mentioned above, in particular, with migraine headaches. In 2014, Burstein et al. were able to demonstrate that BoNTA inhibits mechanical nociception in peripheral trigeminovascular neurons. It is widely accepted that trigeminal and meningeal nociceptors have an important role in the initiation of migraine headaches [17]. Burstein et al. showed that BoNTA prevents the fusion of high-threshold, mechanosensitive ion channels involved with mechanical pain into the nerve terminal membrane, thereby reducing the ability to initiate migraines.

Here, we present the clinical characteristics and treatment response in children with chronic migraines treated with BoNTA at our institutions, Duke and State University of New York (SUNY) Upstate.

## Materials and methods

A retrospective analysis of 30 adolescent migraineurs (25 of whom were in the SUNY Upstate pediatric neurology clinic and five of whom were in the Duke pediatric neurology clinic) who met ICHD-3 criteria for chronic migraine and were treated with Botox (BoNTA; Allergan, Dublin, Ireland) injection according to the standardized adult protocol endorsed by Allergan itself. Patients offered BoNTA treatment had failed two oral preventative medications. Adverse risks with BoNTA were carefully reviewed with patients prior to each injection appointment. Given the increased risk of BoNTA use causing clinically significant effects with pre-existing neuromuscular disorders, a thorough neurologic assessment, and a review of medical history were done prior to the initiation of treatment and each subsequent visit would involve a focused neurologic assessment prior to treatment. Patients were being treated in these clinics for between 12 and 24 months.

The adult protocol involves 31 predefined sites where five units of BoNTA are injected intramuscularly per site and this protocol is based upon the PREEMPT (Phase III Research Evaluating Migraine Prophylaxis Therapy) protocol of which there were 31 standard sites and up to eight additional sites, as needed, for a dose range of 155 units for 31 sites and up to 195 units for a total 39 sites. The median dose of 155 units and the median number of 31 injection sites were consistently observed across all BoNTA sessions and thus Allergan had endorsed the 31 site pattern. In 2010, two, large, placebo-controlled trials, PREEMPT 1 and 2, demonstrated that Botox significantly decreased the severity and frequency of chronic migraine headache [18-19].

The head/neck region is divided into seven specific regions as follows: corrugator receiving 10 units over two sites, procerus receiving five units over one site, frontalis receiving 20 units over four sites, temporalis receiving 40 units over eight sites, occipitalis receiving 30 units over six sites, cervical paraspinal muscles receiving 20 units over four sites, and trapezius receiving 30 units over six sites. This is a total of 155 units. In addition to these standard injection sites, similar to the PREEMPT studies, the pediatric neurology team utilized the concept of additional sites, however, they did not utilize additional sites on an “as-needed basis” but rather administered an additional 30 units definitively. These additional units were given over the chosen six sites with five units administered per site for a total of 185 units. These additional six sites were chosen based upon myofascial pain elicitation via palpation of the head/neck regions and generally were spread out over multiple regions of the seven described above; not all sites were chosen from one region only. 

The decision to use this “hybrid” injection pattern of the adult standard plus a pain-specific additional injection was to optimize potential improvement in headache frequency and intensity. The access data.fda.gov website is clear that FDA approval is for adult chronic migraineurs only, however, given the fact that there have been some small studies utilizing BoNTA in the pediatric populations and at doses not far different, with no significant side effects, the decision had been made to offer BoNTA treatment to these patients in a similar off-label manner.

Single-use, sterile 100 unit vacuum-dried Botox powder vials were reconstituted with sterile, preservative-free 0.9% sodium chloride solution. Injections were solely performed by one pediatric neurologist.

Descriptive statistics and paired t-tests were performed with SPSS v21 (IBM Corp, Armonk, NY, US) in order to process the data and obtain results. Results are reported in a mean ± standard deviation format.

## Results

Participants (n=30) were 16.5 ± 1.83 years old with 24 (80%) of the patients female. The headaches were precipitated by trauma in seven cases. All had failed standard pharmacotherapy, including amitriptyline and topiramate. Results are reported in a mean ± standard deviation format. An average of 2.47 ± 1.6 number of BoNTA injection cycles was performed, ranging from one to five. Migraine severity decreased significantly from 7.47 ± 1.89 on a 10-point visual analog scale (VAS) to 4.34 ± 3.02 (p<.001) and headache frequency from 24.4 ± 7.49 painful days per month to 14.8 ± 12.52 painful days per month (p<.001). One patient developed nausea related to the injection; all others tolerated it well, with no side effects. Table 1 summarizes this.

**Table 1:** Migraine Headache Frequency and Severity Pre- and Post-Botox Administration

	Pre-Botox	Post-Botox	p-value
Headache Frequency	24.4 ± 7.49	14.8 ± 12.52	< .001
Headache Severity	7.47 ± 1.89	4.34 ± 3.02	< .001

**Figure 1:**
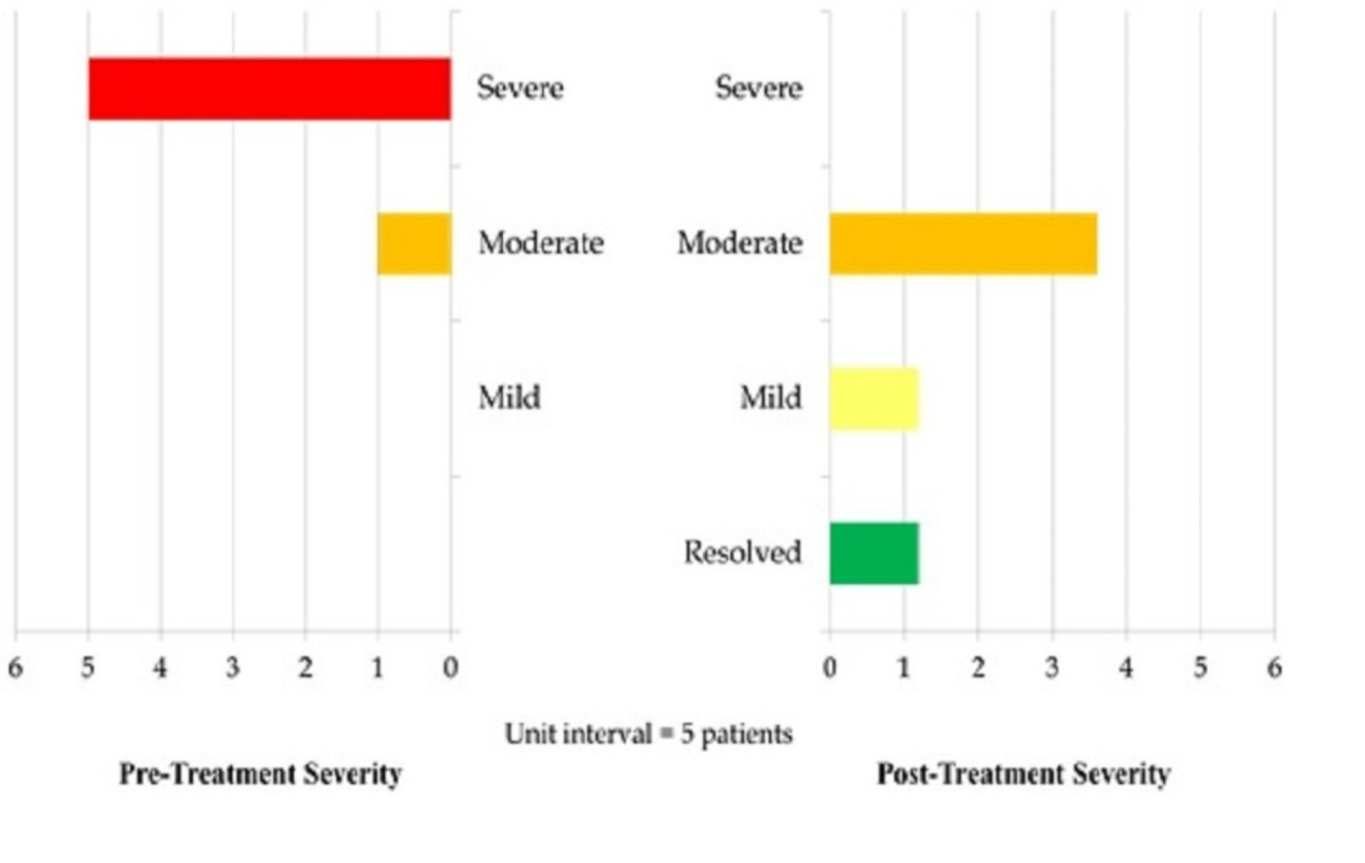
Pre- and Post-Treatment Severity

Headache severity was assessed with the VAS. Scores of 1-3 were considered mild, 4-6 moderate, and 7 and above severe. Out of the 30 patients, the majority had a pre-treatment score falling within the severe category. As visualized in Figure 1, after treatment with Botox, there was not a single patient whose headache remained in the severe range.

## Discussion

Chronic migraine is a serious problem and can be particularly devastating, as it affects school work, extracurricular activities, and quality of life. It commonly leads to missed school days and underperformance in attended school days, which results in a poorer academic outcome. Social development is often affected. Additionally, there is an increased burden on family members, including on parents or caregivers, resulting in decreased relationship quality and increased anxiety and depression. Thus, we are looking at a situation where multiple people are affected [20-23].

There are no FDA-approved medications for chronic migraine in the pediatric population. This, along with the fact that it has been difficult to establish clear headache guidelines for the pediatric population given the lack of randomized controlled trials, shows just how much of a challenge treating chronic migraine in the pediatric population is. The American Academy of Neurology, French Society for the Study of Migraine and Headache, Italian Society for the Study of Headache, and International Headache Society have all been involved in producing various guidelines [24].

Although the pathomechanism of migraine is not fully understood, there are treatments that have been effective in the adult population that are not even specific for migraine such as antidepressants, antiepileptics, antihistamines, calcium ion channel antagonists, and beta-adrenergic receptor blockers. These are also being utilized in the pediatric population, however, on a smaller scale, as data from adult studies are often extrapolated and applied to the pediatric population as well [25-26]. There have been mixed results with this approach for pediatric patients who do receive such treatment, and although it appears that many of these preventative medications are safe, there is a concern that pharmacological interventions may have the potential to present more risk than benefits in this population and, therefore, despite experiencing significant disability, a large number of pediatric migraineurs do not receive prophylactic therapy [25]. There is strong evidence throughout the literature that personalized therapy tailored to the pediatric population, including children and adolescents, is the best approach, with a strong component being cognitive behavioral therapy [14,27]. According to one retrospective study reviewing data over an 11-year time frame at the Cincinnati Children's Hospital Headache Center, the majority of pediatric patients who received multimodal interdisciplinary care for migraines improved over time [28].

While we are awaiting pharmaceutical innovation in the development of pathomechanism-based antimigraine drugs, the multimodal approach appears to be the way to go. Given the mixed results of oral medications and the concern of potential harm, although there has yet to be any major concerns with any one particular medication and given the significant results of onabotulinum toxin type A injections, including our retrospective data, perhaps patients already on oral medications can be weaned off some or all of these medications while receiving onabotulinum toxin. Despite our lack of complete understanding of the pathomechanism(s) of migraine, we at least have a basic understanding of how this toxin works in migraine as opposed to some of the oral medications. Importantly, there have been limited side effects of the use of onabotulinum in the adult migraine population and among not only our retrospective study but with the other retrospective studies as well that have investigated the efficacy of onabotulinum toxin use; we had good treatment tolerance in our group. According to one study, patients had attempted an average of eight therapies prior to onabotulinum type A use and most patients reported adverse events from at least one of these prior medications [13]. This suggests again that oral prophylaxis has not been very convincing and easier to have adverse events in the pediatric population.

There is a lot to gain for the pediatric population with further research into onabotulinum toxin use. It would be interesting to see if there is a certain chronic migraine profile that would suggest that onabotulinum toxin would be more likely to succeed and if this treatment would be efficacious in patients with migraine variants.

How the pediatric chronic migraine multimodal prevention model will look in the near future is the big question. Perhaps ultimately, we will evolve to a model with cognitive behavioral therapy (CBT) and onabotulinum toxin use as the central pillars, plus or minus an oral medication from one of the current classes of medications for prophylaxis if onabotulinum is partially efficacious and the model may also include certain non-pharmacological interventions. Another question to ask is what will the role of cGRP inhibitors be in pediatric migraine prophylaxis, as it is widely utilized now in the adult migraine population. Perhaps there may be an additive effect of cGRP inhibitors and onabotulinum toxins to further improve headache frequency and severity, and it may be utilized in situations where onabotulinum toxin is partially efficacious but not fully adequate alone. In these cases, the cGRP inhibitors may be utilized as adjunct medication alongside onabotulinum toxin. Additionally, can there be one true multimodal model for the pediatric population as a whole or will we eventually have a model A and model B with differences for children vs adolescents? In our retrospective data, patients were mostly adolescents and girls, therefore, applicability to younger patients and males may be limited. What about the younger children in terms of onabotulinum use? Do we need pilot studies for younger children and Botox use and randomized controlled trials (RCTs) for the adolescents, as we already have good pilot data or do we just keep children and adolescents in the same category and move on with RCTs?

The placebo response has been greater in pediatric as compared to adult patients, as there has been a reduction in the attack frequency in the absence of any pharmacologic treatment more frequently observed in the pediatric population. The shorter duration of migraine attacks and other semeiological features of migraines in children have made it difficult to design adequate randomized control trials. Also, children encounter difficulty in describing the features of headache and any associated symptoms [10,29]. Although the issues above are making it difficult to design proper randomized controlled trials, the significant improvement seen with our retrospective data and the prior onabotulinum studies in the pediatric population clearly demonstrate that the time is now for taking this next step and designing such trials.

## Conclusions

BoNTA injection was a safe and effective therapy for chronic migraine in our cohort of children recalcitrant to medical therapy. Further research with multicentered, double-blinded, randomized, placebo-controlled trials is warranted to evaluate the long-term safety and efficacy in this population. There are small retrospective studies already published, commenting on different aspects, such as chronic migraine frequency, intensity, duration, and effect on quality of life, to name a few. This study further suggests that BoNTA is both safe and effective in the pediatric population. Further, prospective placebo-controlled randomized research is necessary to definitively to explain the role of BoNTA injection in pediatric chronic migraineurs.
